# Preoperative prediction of meningioma grade based on synthetic MRI quantitative T1 and T2 mapping

**DOI:** 10.1007/s11604-026-02014-5

**Published:** 2026-05-25

**Authors:** Tsubasa Nakano, Junki Kamizono, Tomohito Hasegawa, Masanori Nakajo, Kiyohisa Kamimura, Ryota Nakanosono, Hiroaki Nagano, Koji Takumi, Ryoji Yamagishi, Fumitaka Ejima, Fumiko Kanzaki, Nayuta Higa, Hajime Yonezawa, Mari Kirishima, Ikumi Kitazono, Takashi Yoshiura

**Affiliations:** 1 Department of Radiology, Kagoshima University Graduate School of Medical and Dental Sciences, 8-35-1 Sakuragaoka, Kagoshima, 890-8544 Japan; 2 Department of Advanced Radiological Imaging, Kagoshima University Graduate School of Medical and Dental Sciences, 8-35-1 Sakuragaoka, Kagoshima, 890-8544 Japan; 3 Department of Neurosurgery, Kagoshima University Graduate School of Medical and Dental Sciences, 8-35-1 Sakuragaoka, Kagoshima, 890-8544 Japan; 4 Department of Pathology, Kagoshima University Graduate School of Medical and Dental Sciences, 8-35-1 Sakuragaoka, Kagoshima, 890-8544 Japan

**Keywords:** Synthetic MRI, Meningioma, Tumor grading, T1 map, T2 map, Quantification

## Abstract

**Purpose:**

This study aimed to investigate the difference in T1, T2, and proton density (PD) values derived from synthetic magnetic resonance imaging (MRI) across various meningioma subtypes and their potential as imaging biomarkers for the World Health Organization (WHO) grade classification of meningioma.

**Materials and methods:**

This study retrospectively analyzed the data of 100 consecutive meningioma cases. Across subtypes and WHO grades, intratumoral T1, T2, PD, and apparent diffusion coefficient (ADC) values, along with tumor volume, were measured and compared, and the discriminative performance of each parameter was evaluated using the area under the receiver operating characteristic curve (AUC). Simple and multiple regression analyses were conducted to assess the associations between quantitative imaging parameters and atypical pathological features (4 ≥ mitotic findings/high power field; brain invasion; increased cellularity; small cells with high nuclear-to-cytoplasmic ratio; prominent nucleoli; sheeting; necrosis).

**Results:**

Among all subtypes, angiomatous, microcystic, and metaplastic meningioma demonstrated remarkable high T1 and T2 values. The AUCs for differentiating these subtypes from other subtypes were 0.97 for T1 value and 0.99 for T2 value. Volume (*p* < 0.001), T1 value (*p* < 0.001), T2 value (*p* = 0.002), and ADC value (*p* = 0.035) were significantly higher in high grade meningioma than low grade meningioma, with an AUC of 0.83 achieved by combining volume, T1, and ADC value. Multiple regression analysis revealed that volume was significantly associated with necrosis (*p* = 0.018), whereas T1 and T2 values were significantly associated with sheeting (*p* = 0.047 and 0.025 for T1 and T2).

**Conclusion:**

T1 and T2 mappings may serve as useful quantitative imaging biomarkers for meningioma grading.

**Supplementary Information:**

The online version contains supplementary material available at https://doi.org/10.1007/s11604-026-02014-5.

## Introduction

Meningioma is the most common intracranial tumor in adults [1]. According to the latest World Health Organization Central Nervous System classification of 2021, meningioma is classified as World Health Organization (WHO) grades 1, 2, or 3, and their biological behavior substantially varies among tumors [2]. Low-grade meningioma (LGM, WHO grade 1) has lower proliferative activity, and active surveillance is considered one of the management options [3]. With adequate surgical resection, the recurrence rate remains low, at approximately 20% in 20 years [4]. Conversely, high-grade meningioma (HGM, WHO grade 2 or 3) frequently requires adjuvant radiotherapy postoperatively due to its higher risk of recurrence at 41% and 75% for WHO grades 2 and 3 meningioma, respectively [5, 6]. Therefore, predicting the WHO grade is highly important for identifying treatment strategies for meningioma.

Imaging, particularly magnetic resonance imaging (MRI), is the most effective tool for the preoperative diagnosis of meningioma. Several studies have attempted to classify WHO grades according to MRI findings, and various factors, including tumor morphology, location, signal intensity, and contrast enhancement, are indicators associated with tumor grade [7–10]. However, the integration of these imaging findings does not always facilitate straightforward classification, and the identification of novel biomarkers is continuously warranted.

Synthetic MRI (SyMRI) is a recently developed MRI technique that has been increasingly adopted in clinical practice. Unlike conventional MRI techniques, SyMRI quantifies tissue T1, T2, and proton density (PD) values and synthesizes various images, including T1-weighted image (T1WI), T2-weighted image (T2WI), fluid attenuated inversion recovery, short tau inversion recovery, and double inversion recovery, from these quantitative parameters [11]. This is a remarkable technique that allows the acquisition of multiple contrast-weighted images and quantitative maps in a single scan. These quantitative parameters were applied to tumor diagnosis, and several studies have revealed their usefulness in differentiating benign and malignant lesions or tumor grading [12]. However, multiparametric quantitative evaluation with SyMRI for meningioma grading has not been previously reported. Our primary aim is to investigate the differences in T1 and T2 values according to the WHO grade of meningioma and to evaluate their usefulness for preoperative grading. A secondary aim is to compare T1 and T2 values with pathological features in order to elucidate which tissue characteristics underlie the differences in T1 and T2 values.

## Materials and methods

### Study population

Our Institutional Review Board approved this retrospective study and waived the need for informed consent, considering the retrospective nature of this study. This study included consecutive patients with a pathological diagnosis of meningioma from April 2021 to September 2025. Lack of SyMRI data was the only exclusion criterion.

### Image acquisition and processing

All MRIs were acquired with a 3-T system (Achieva dStream, Philips) using a 20-channel head coil. We employed a two-dimensional axial method with a QURAPMASTER (quantification of relaxation times and PD by multi-echo acquisition of saturation-recovery using turbo spin echo readout) pulse sequence to acquire synthetic MRI. Two echo times (TE, 12/50 ms) and four delay times (110/440/1210/2530 ms) were used to generate 8 actual images and 8 imaginary images. Other parameters include repetition time (TR), 5000 ms; flip angle, 90°; acceleration factor, 3; field of view (FOV), 230 × 230 mm^2^; matrix size, 512 × 512; slice thickness, 5 mm; slice gap, 1 mm; number of slices, 24; acquisition time, 330 s. Further, we used conventional three-dimensional (3D) contrast-enhanced T1WI (CE-T1WI), apparent diffusion coefficient (ADC) map derived from diffusion weighted image (DWI), 2D axial plane T1WI, and 2D axial plane T2WI for tumor volume calculation, ADC value measurement, and qualitative tumor signal intensity analysis. The scan parameters were as follows for CE-T1WI (TE, 3.79 ms; TR, 8.14 ms; inversion time, 1044; flip angle, 8°; acceleration factor, 3; FOV, 240 × 240 mm^2^; matrix size, 480 × 480; slice thickness, 2 mm; number of slices, 80; acquisition time, 170 s), DWI (TE, 80 ms; TR, 4600 ms; flip angle, 90°; acceleration factor, 3; b-values, 0, 1000 s/ mm^2^; diffusion gradient direction, 3; FOV, 230 × 230 mm^2^; matrix size, 256 × 256; slice thickness, 5 mm; slice gap, 1 mm; number of slices, 24; acquisition time, 56 s), T1WI (TE, 400 ms; TR, 10 ms; flip angle, 90°; FOV, 230 × 230 mm^2^; matrix size, 512 × 512; slice thickness, 5 mm; slice gap, 1 mm; number of slices, 24; acquisition time, 151 s), and T2WI (TE, 4000 ms; TR, 80 ms; flip angle, 90°; FOV, 230 × 230 mm^2^; matrix size, 512 × 512; slice thickness, 5 mm; slice gap, 1 mm; number of slices, 24; acquisition time, 104 s).

### Quantitative analysis

A freehand region of interest (ROI) was manually drawn on the axial slice of CE-T1WI, demonstrating the largest cross-sectional area of the tumor and avoiding obvious cyst or necrosis. Calcification was not excluded. The generated ROIs were copied onto the T1, T2, PD and ADC maps, and the mean values were calculated. Further, the tumor volume was calculated using the ABC/2 formula [13] on CE-T1WI (A, maximum diameter on the axial plane; B, diameter perpendicular to A; C, maximum height on the coronal/sagittal plane). Two radiologists with 10 and 20 years of radiology experience who were blinded to the clinical and pathological information performed the segmentation and volume calculation process on a medical imaging system (SYNAPSE SAI viewer version 2.2, Fujifilm Medical, Tokyo, Japan). Two radiologists provided the averaged values of the measurements, which were used for quantitative analysis.

### Qualitative analysis

Elster criteria [14] was used to evaluate tumor signal intensity scores as follows on T1WI: 1, significantly hypointense compared with gray matter, close to cerebrospinal fluid (CSF); 2, slightly hypointense compared with gray matter; 3, isointense compared with gray matter; 4, slightly hyperintense compared with gray matter; 5, significantly hyperintense compared with gray matter, close to fat. Similarly, as follows on T2WI: 1, significantly hypointense compared with gray matter, close to bone cortex; 2, slightly hypointense compared with gray matter; 3, isointense compared with gray matter; 4, slightly hyperintense compared with gray matter; 5, significantly hyperintense compared with gray matter, close to CSF.

### Histopathological diagnosis

Histopathological diagnosis was established based on the WHO classification of tumors 2021 for all patients. Along with the tumor subtype and WHO grade, the presence or absence of pathological findings incorporated in the diagnostic criteria for atypical meningioma was recorded. The pathological findings included ≥ 4 mitoses per high power field; brain invasion; increased cellularity; small cells with high nuclear-to-cytoplasmic (N: C) ratio; prominent nucleoli; sheeting; necrosis.

### Statistical analysis

The intraclass correlation coefficient (ICC) was used to evaluate interobserver agreement (ICC = 0.00–0.20, poor correlation; ICC = 0.21–0.40, fair correlation; ICC = 0.41–0.60, moderate correlation; ICC = 0.61–0.80, good correlation; ICC = 0.81–1.00, excellent correlation). The Mann–Whitney U test was used to compare the quantitative values between the two groups. The area under the curve of the receiver operating characteristic analysis was used to evaluate diagnostic performance of each quantitative parameter. The chi-square test was conducted to compare signal intensity scores on conventional images between LGM and HGM. Simple and multiple regression analyses were conducted to assess the associations between quantitative imaging parameters and atypical pathological findings. MedCalc (version 22.4.0, MedCalc) was used for all statistical analyses. *P-*values of < 0.05 indicated statistical significance.

## Results

After reviewing our institutional imaging database and clinical records, we identified 147 pathologically diagnosed patients with meningioma from April 2021 to September 2025. Of these, 47 patients without synthetic MRI data were excluded. No additional patients were excluded for other reasons, and the final study cohort comprised 100 patients.

ICCs were excellent for all quantitative parameters (volume, 0.95; T1, 0.99; T2, 0.99; PD, 0.94; ADC, 0.95). Conversely, ICCs of signal intensity scores were lower than those of quantitative parameters (T1WI, 0.39; T2WI, 0.66).

Table 1 summarizes tumor volume, SyMRI-based quantitative values, and ADC values for all subtypes. Angiomatous, microcystic, and metaplastic meningioma demonstrated remarkably high T1 and T2 values among all subtypes (Supplementary Figure S1, Supplementary Table S1). The AUCs for differentiating these subtypes from other subtypes were 0.97 (95% confidence interval [CI]: 0.92–0.99) for the T1 value and 0.99 (95% CI: 0.95–1) for the T2 value (Supplementary Figure. S2). After excluding cases with markedly increased T2 values (cutoff: >127.1 ms), subsequent analyses were conducted according to WHO grade.

**Table 1:** Tumor volume, SyMRI-based T1, T2, PD values, and ADC values for all subtypes

	Volume (cm^3^)	T1 (ms)	T2 (ms)	PD (%)	ADC (10^− 3^mm^2^/s)
*WHO grade 1*					
Meningothelial (*n* = 32)	12.3 ± 16.3	1385.1 ± 109.4	87.7 ± 9.8	90.7 ± 6.3	859.4 ± 118.1
Fibrous (*n* = 4)	19.6 ± 24.4	1210.4 ± 180.5	73.1 ± 16	83.1 ± 7.2	838.4 ± 64.2
Transitional (*n* = 2)	76.8 ± 62.4	1540.3 ± 246.7	101.5 ± 17.6	87.5 ± 0.6	848.6 ± 96.8
Angiomatous (*n* = 2)	4.9 ± 0.3	2593.4 ± 251.6	177.2 ± 40.8	92.8 ± 0.4	1457.7 ± 393.2
Microcystic (*n* = 2)	21.6 ± 25.8	2246.7 ± 459.3	176.4 ± 37.7	91.7 ± 1.5	1072.6 ± 185.5
Metaplastic (*n* = 1)	5	1960.2	171.4	89.3	1402
*WHO grade 2*					
Atypical (*n* = 53)	37.3 ± 32.8	1535.9 ± 301.3	96.7 ± 21.2	89.7 ± 4.1	832.7 ± 154.2
Chordoid (*n* = 2)	13.7 ± 7.2	1468.5 ± 145.1	99.3 ± 19.4	92.1 ± 3.5	865.6 ± 81.3
WHO grade 3					
Anaplastic (*n* = 2)	72.5 ± 55.8	1521.6 ± 26.6	95.9 ± 4.4	87.2 ± 0.8	759.1 ± 10.1

Values are expressed as mean ± standard deviation. PD, Proton density; ADC, Apparent diffusion coefficient

Table 2 summarizes demographic data and basic tumor characteristics of the patients. Patients with HGM were significantly older and had a higher proportion of males compared with those with LGM. In addition, the frequency of calcification was significantly higher in HGM than LGM.

**Table 2:** Comparison of demographic data and basic tumor characteristics between low grade meningioma and high grade meningioma

	LGM (*n* = 38)	HGM (*n* = 55)	*P* value
Age	58.9 ± 13.4	63.9 ± 17.6	0.041
Sex			< 0.001
Male	2	20	
Female	36	35	
Location			0.613
Convexity/falx/parasagittal	18	29	
Others	20	26	
Calcification			0.013
Positive	10	29	
Negative	24	22	

LGM, Low grade meningioma (WHO grade 1); HGM, High grade meningioma (HGM, WHO grade 2 or 3)

**Fig. 1:**
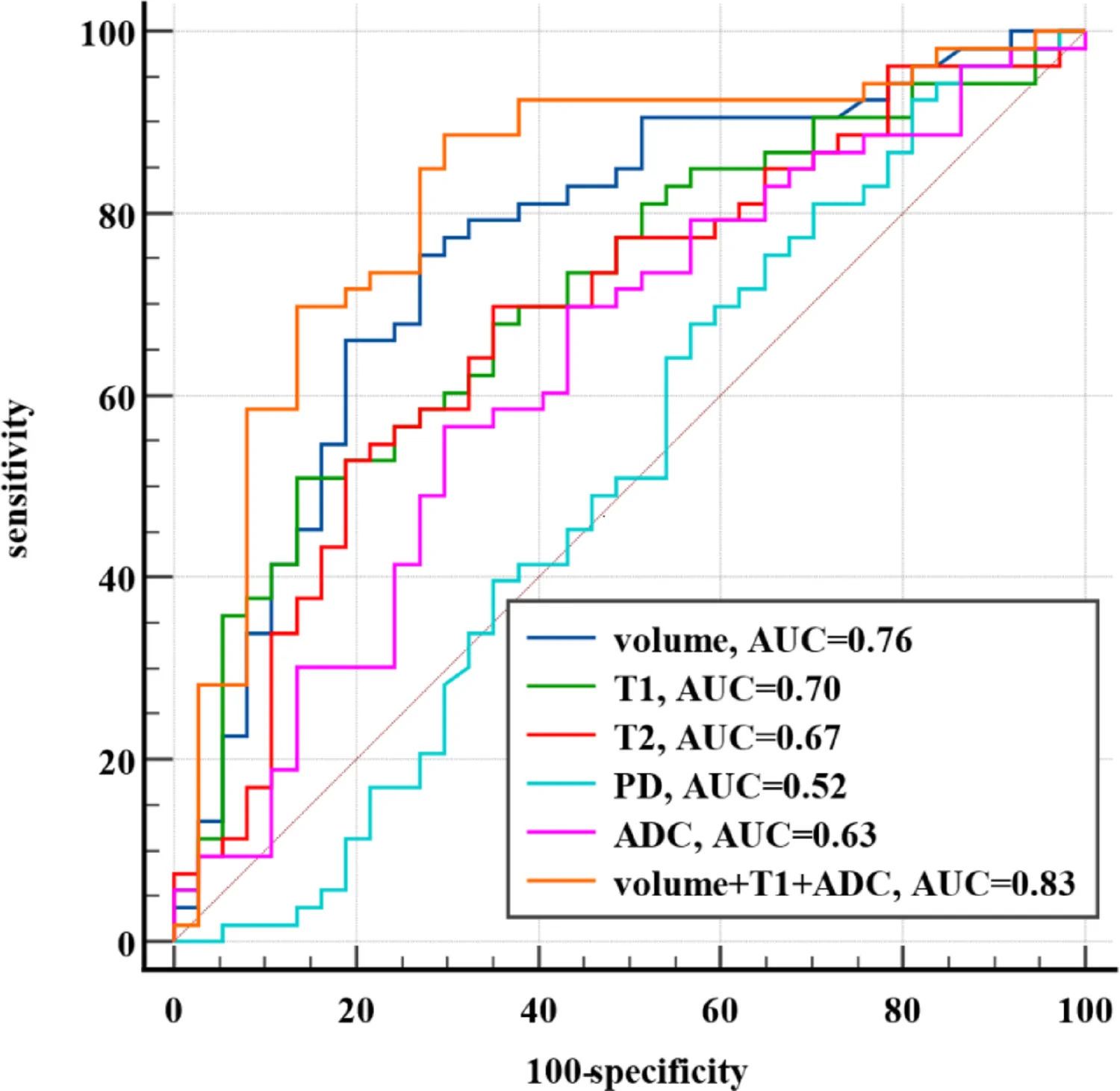
Receiver operating characteristic curves for discrimination between low grade meningioma and high grade meningioma

The comparison of LGM and HGM revealed significantly higher volume (*p* < 0.001), T1 value (*p* < 0.001), and T2 value (*p* = 0.005), and lower ADC value (*p* = 0.035) in HGM, whereas PD (*p* = 0.757) demonstrated no significant difference between LGM and HGM. (Table 3). The AUCs were 0.76 (95% CI: 0.67–0.85) for volume, 0.70 (95% CI: 0.6–0.79) for T1 value, 0.67 (95% CI: 0.57–0.77) for T2 value, and 0.63 (95% CI: 0.52–0.73) for ADC value for differentiation between LGM and HGM. The combination of volume, T1 value and ADC value generated a higher AUC of 0.83 (95% CI: 0.73–0.90) (Fig. 1).

**Table 3:** Comparison of quantitative values between low grade meningioma and high grade meningioma

	LGM	HGM	*P* value	Cutoff	Sensitivity (%)	Specificity (%)	Accuracy (%)
Volume (cm^3^)	16.4 ± 24.2	38.2 ± 33.9	< 0.001	> 14.8	74.5	73.7	76.7
T1 (ms)	1374.8 ± 137.1	1490.7 ± 188.5	< 0.001	> 1495.2	49.1	86.8	64.5
T2 (ms)	86.9 ± 11.9	93.8 ± 13.5	0.005	> 89.1	67.3	65.8	66.7
PD (%)	89.7 ± 6.5	89.6 ± 4	0.757	≤ 91.06	69.1	44.7	59.1
ADC (10^− 3^mm^2^/s)	856.5 ± 110.8	812.4 ± 94.8	0.035	≤ 806.94	56.6	70.3	62.2

Values are expressed as mean ± standard deviation. PD, Proton density; ADC, Apparent diffusion coefficient; LGM, Low grade meningioma (WHO grade 1); HGM, High grade meningioma (HGM, WHO grade 2 or 3)

The qualitative analysis of signal intensity scores revealed that T1WI scores significantly differed between LGM and HGM for reader 1, whereas no significant difference was observed in the scores of reader 2. T2WI scores demonstrated no significant differences between LGM and HGM for either reader (Table 4).

**Table 4:** Comparison of signal intensity scores between low grade meningioma and high grade meningioma

	Reader 1			Reader 2		
	LGM	HGM	*P* value	LGM	HGM	*P* value
T1WI score			0.042			0.122
1	0(0)	1(1)		0(0)	0(0)	
2	2(5)	10(17)		15(39)	25(43)	
3	36(95)	41(72)		13(34)	26(45)	
4	0(0)	5(9)		10(26)	6(10)	
5	0(0)	0(0)		0(0)	0(0)	
T2WI score			0.328			0.399
1	1(3)	0(0)		0(0)	0(0)	
2	1(3)	2(3)		2(5)	3(5)	
3	12(31)	11(19)		4(10)	2(3)	
4	24(63)	43(75)		32(84)	51(89)	
5	0(0)	0(0)		0(0)	0(0)	

Values in parentheses indicate percentiles. LGM, Low grade meningioma (WHO grade 1); HGM, High grade meningioma (WHO grade 2 or 3)

Table 5 summarizes the results of simple and multiple regression analyses assessing the associations between quantitative imaging parameters and atypical pathological findings. The simple regression analysis revealed that volume demonstrated significant differences for small cells with a high N: C ratio, necrosis, and sheeting. However, the multiple regression analysis identified that only necrosis remained significant. Increased cellularity, small cells with a high N: C ratio, prominent nucleoli, and sheeting demonstrated significant differences in the simple regression analysis for T1 and T2 values; however, the multiple regression analysis identified that only sheeting remained significant. For ADC, only necrosis showed a significant difference in the simple regression; therefore, multiple regression was not performed.

**Table 5:** Simple and Multiple regression analyses assessing relationships between quantitative imaging parameters and atypical pathological findings

	Negative	Positive	*P* value	
			Simple regression	Multiple regression
*Volume*				
Mitosis ≥ 4	28.3 ± 32.4	33.0 ± 29.4	0.563	
Increased cellularity	16.9 ± 30.8	31.5 ± 31.5	0.102	
Small cells with high N: C ratio	18.0 ± 25.5	35.1 ± 33.3	0.012	0.242
Prominent nucleoli	26.9 ± 29.3	44.8 ± 43.7	0.068	
Necrosis	23.7 ± 29.1	45.6 ± 32.6	0.003	0.018
Sheeting	22.5 ± 29.5	36.3 ± 32.8	0.034	0.486
Brain invasion	27.6 ± 31.8	43.2 ± 29	0.141	
*T1*				
Mitosis ≥ 4	1463.9 ± 227.1	1493.2 ± 344.7	0.655	
Increased cellularity	1351.1 ± 161.5	1492 ± 261.4	0.047	0.816
Small cells with high N: C ratio	1360.1 ± 134.6	1528.1 ± 281.4	0.002	0.342
Prominent nucleoli	1447.4 ± 207.6	1624.5 ± 442.7	0.023	0.149
Necrosis	1443.6 ± 226.8	1547.1 ± 310.9	0.08	
Sheeting	1384.5 ± 139.6	1560.6 ± 310.7	< 0.001	0.047
Brain invasion	1472.8 ± 261.2	1444.2 ± 176	0.738	
*T2*				
Mitosis ≥ 4	92.5 ± 17	94.1 ± 23.1	0.741	
Increased cellularity	83.1 ± 14.4	94.6 ± 18.3	0.024	0.439
Small cells with high N: C ratio	85.7 ± 11.9	96.6 ± 19.9	0.005	0.529
Prominent nucleoli	92.1 ± 17.2	98 ± 24.6	0.291	
Necrosis	92.3 ± 17.7	94.2 ± 20.1	0.666	
Sheeting	86.6 ± 11.6	99.3 ± 21.6	< 0.001	0.025
Brain invasion	93.3 ± 18.5	88.9 ± 16.3	0.478	
*ADC*				
Mitosis ≥ 4			0.309	
Increased cellularity			0.118	
Small cells with high N: C ratio			0.243	
Prominent nucleoli			0.979	
Necrosis			0.02	
Sheeting			0.613	
Brain invasion			0.155	

Values are expressed as mean ± standard deviation. ADC, Apparent diffusion coefficient

**Fig. 2:**
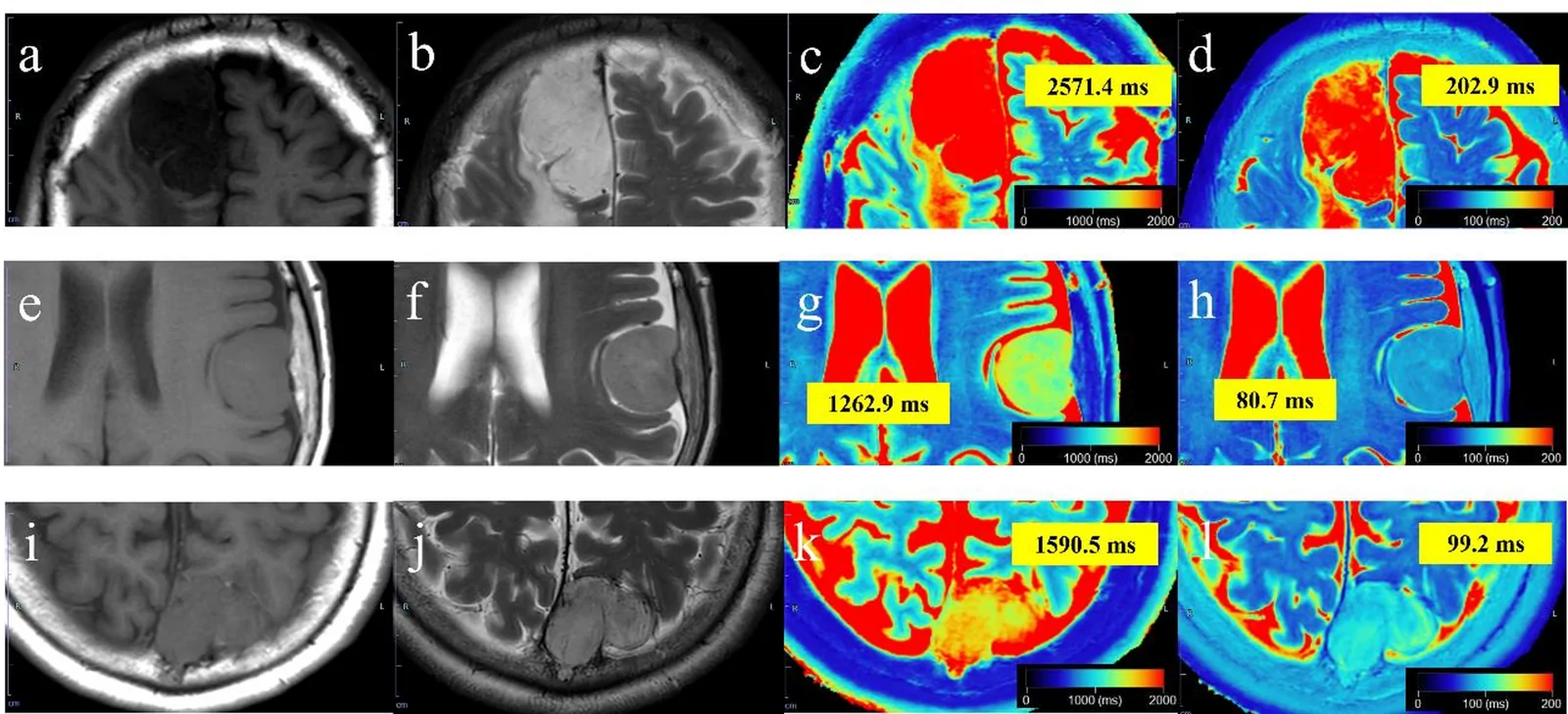
Upper row: a 64-year-old male patient with microcystic meningioma. No atypical pathological features were observed. The tumor demonstrated markedly hypointense on T1 weighted image (**a**) and markedly hyperintense on T2 weighted image (**b**), with correspondingly high values on T1 map (**c**) and T2 map (**d**). Middle row: a 66-year-old female patient with meningothelial meningioma (**e**), T1 weighted image; **f**, T2 weighted image; **g**, T1 map; **h**, T2 map). An increased cellularity was the only atypical pathological finding observed. Lower row: a 71-year-old female patient with atypical meningioma. Increased cellularity, small cells with high N/C ratio, and sheeting were observed. Although the tumor showed no marked differences from meningothelial meningioma on T1 weighted image (**i**) and T2 weighted image (**j**), it demonstrated distinctly higher values on the T1 map (**k**) and T2 map (**l**)

Figure 2 illustrates the images of representative cases with different subtypes of meningioma.

## Discussion

Among the many subtypes of meningioma, some demonstrate highly characteristic signal intensity on MRI [15]. The present quantitative analysis revealed that angiomatous/microcystic/metaplastic meningioma showed markedly higher T1 and T2 values than other meningiomas, enabling easy differentiation. However, these rare subtypes are only a very small fraction of meningioma, and the real issue lies with the majority of others.

Several studies regarding MRI signal intensity have reported that high signal intensity on T2WI is associated with higher WHO grade or rapid tumor growth of meningioma [12, 16–18]. Conversely, a meta-analysis revealed that the signal intensity on T2WI does not differ among meningioma grades, and the results differed across studies [8]. Most of these studies investigated whether meningioma demonstrated higher or lower signal intensity compared with normal cerebral gray matter. We performed a similar qualitative assessment but reported no favorable results. The signal contrast on T2WI is influenced by image acquisition parameters as well as window width and level settings; thus, such variations in results are considered inevitable. Moreover, in our results using Elster criteria, both readers classified the T2WI scores as 4 in the majority of the cases. This indicates that the signal intensity of meningioma on T2WI mostly lies between cerebral gray matter and CSF, and the current evaluation method may not be sensitive enough to detect subtle differences in the signal intensity of meningioma. In T1WI, reader 1 classified most cases as score 3, whereas reader 2 classified a certain number of cases as score 2 or 4. Hence, the interobserver agreement became much lower than that of T2WI. The signal intensity of meningioma and cerebral gray matter is very similar on T1WI, with some difficulty in assigning the scores, which may cause greater interobserver variability.

Unlike the qualitative analysis, our quantitative analysis revealed that both T1 and T2 values were higher in HGM than in LGM. Quantification likely enabled a more sensitive detection of subtle changes within the tumor microenvironment associated with malignant transformation. By combining the T1 value with tumor volume and ADC value, which are well-known risk factors for HGM [8, 12, 19–21], we achieved high discriminatory ability. Further, the interobserver agreement was very high, supporting the reliability of these quantitative values. Meningioma is generally characterized by well-defined margins and homogeneous tumors; thus, measurement variability is less likely, making it well-suited for quantitative evaluations.

**Fig. 3:**
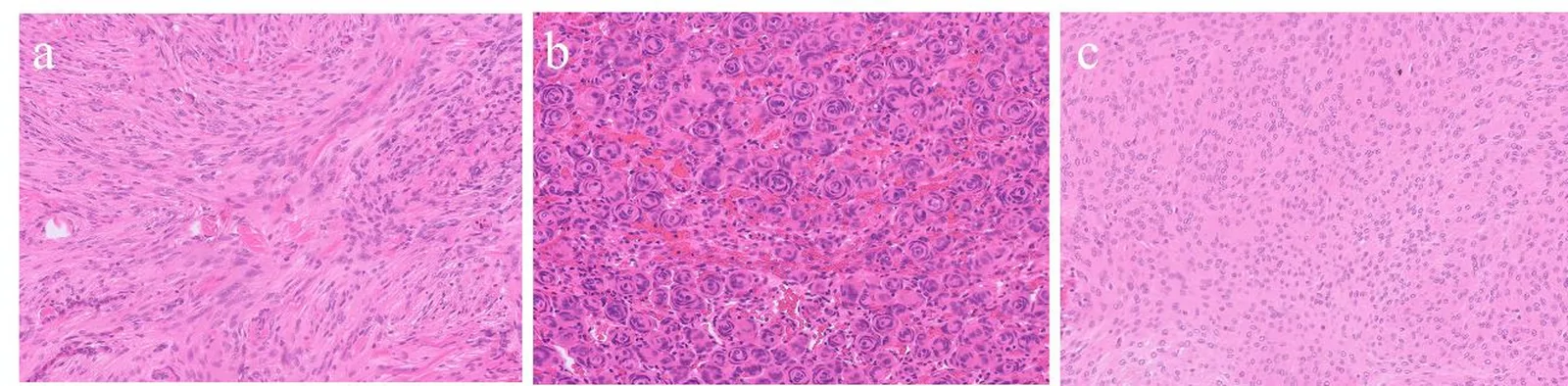
Growth patterns of meningioma observed on hematoxylin and eosin-staining. Fascicular pattern (**a**), whorled pattern (**b**), sheet-like pattern (**c**).

Cao et al. [21] reported that the T1 value of meningioma was higher in HGM compared with LGM. Our findings were consistent with their results. Other than meningioma, similar results have been reported in rectal [22], renal [23], and breast [24] lesions. However, several studies have revealed opposite findings, demonstrating that higher malignancy is associated with lower T1 values in salivary gland [25], nasopharyngeal [26], thyroid [27], liver [28], and prostate [29] lesions. Regarding T2 values, this is the first study to show an association between T2 values and meningioma grade. As mentioned above, considering that several studies have reported findings associated with signal intensity on T2WI, this result is considered expected; however, similar to T1, several reports describe the opposite trend in other organs, including the liver [30], breast [24], and prostate [31]. Thus, the association between tumor malignancy and T1 or T2 values shows no consistent trend, and this likely depends on the organ and tumor type. In meningioma, T1 and T2 values are considered to increase with tumor grade according to the results of the present study as well as previous reports. Furthermore, our multivariate analysis suggested that the specific atypical pathological feature of sheeting is associated with elevated T1 and T2 values in HGM. Sheeting refers to an uninterrupted patternless or sheet-like growth of tumor cells without forming the typical lobular, whorled, or fascicular structures observed in benign meningioma (Fig. 3). As meningioma becomes more atypical, several biological changes occur, including degeneration and reconstruction of the extracellular matrix (ECM) and reduction of intercellular adhesion and contact inhibition [32]. Sheeting may originate from these changes in the tumor microenvironment. Increases in T1 and T2 values due to ECM degeneration have been proposed in articular cartilage degeneration [33, 34]. Similarly, the increased T1 and T2 values associated with sheeting in HGM observed in this study may be associated with underlying ECM degeneration.

Calcification may also be considered a factor influencing T1 and T2 values of meningioma. Calcification is often observed in meningioma and is known to shorten T1 and T2. However, the frequency of calcification in meningioma has been reported to be higher in HGM than LGM [8], and in our cohort as well, calcification was more frequently observed in HGM. Therefore, calcification is unlikely to be directly related to the present results.

Our study has several limitations. First, it is a retrospective single-center study and included a limited number of cases. Therefore, rare subtypes were either not included in the cohort or, even if included, were difficult to analyze as independent groups. This may lead to an overestimation of the results. Validation utilizing external data would make our results more convincing. Second, the present study included only surgically resected cases. Surgical resection is not always the option for meningioma. In some cases, they are managed with observation depending on the clinical situation. In such cases, further studies that investigate whether T1 and T2 values could serve as predictive factors for meningioma growth are warranted, and they may enable planning more tailored follow-up strategies. Third, the quantitative maps used in this study were 2D images with a 5-mm slice thickness; thus, we conducted a quantitative analysis utilizing only a single slice image. This may not be a major issue since meningiomas are frequently homogeneous in their internal characteristics; however, assessing the entire tumor could potentially provide new insights. Recently, 3D acquisition SyMRI has been introduced [35], and further studies employing this technique are considered a future research prospect.

## Conclusion

T1 and T2 values obtained from SyMRI were higher in HGM than in LGM and demonstrated high diagnostic performance when combined with established quantitative imaging parameters such as tumor volume and ADC values, particularly after excluding rare subtypes with markedly high T2 value, such as angiomatous, microcystic, and metaplastic meningiomas. Furthermore, multivariate analysis suggested that the elevated T1 and T2 values observed in HGM may be associated with the atypical pathological feature of sheeting. We believe that T1 and T2 values can serve as useful imaging biomarkers for preoperative grading of meningioma and may aid in treatment selection.

## Supplementary Information

Below is the link to the electronic supplementary material.


Supplementary Material 1


## Data Availability

The data that support the findings of this study are not openly available and are available from the corresponding author upon reasonable request.

## References

[1] Whittle IR, Smith C, Navoo P, Collie D, Meningiomas. Lancet. 2004;363:1535–43. 15135603 10.1016/S0140-6736(04)16153-9

[2] Louis DN, Perry A, Wesseling P, Brat DJ, Cree IA, Branger-Figarella D, et al. The 2021 WHO classification of tumors of the central nervous system: a summary. Neuro Oncol. 2021;23:1231–51. 34185076 10.1093/neuonc/noab106 PMC8328013

[3] Hallak H, Mantziaris G, Pikis S, Islim AI, Peker S, Samanci Y et al. A retrospective comparison of active surveillance to stereotactic radiosurgery for the management of elderly patients with an incidental meningioma. Acta Neurochir (Wien). 2025:167:37. 10.1007/s00701-025-06452-4 PMC11802698 39912992

[4] Ryu HS, Moon KS, Lee KH, Jang WY, Jung TY, Kim IY, et al. Recurred intracranial meningioma: a retrospective analysis for treatment outcome and prognostic factor. Brain Tumor Res Treat. 2017;5:54–63. 29188205 10.14791/btrt.2017.5.2.54 PMC5700028

[5] Aghi MK, Carter BS, Cosgrove GR, Ojemann RG, Amin-Hanjani S, Martuza R, et al. Long-term recurrence rates of atypical meningiomas after gross total resection with or without postoperative adjuvant radiation. Neurosurgery. 2009;64:56–60. 19145156 10.1227/01.NEU.0000330399.55586.63

[6] Yang S-Y, Park C-K, Park S-H, Kim DG, Chung YS, Jung H-W. Atypical and anaplastic meningiomas: prognostic implications of clinicopathological features. J Neurol Neurosurg Psychiatry. 2008;79:574–80. 17766430 10.1136/jnnp.2007.121582

[7] Yu J, Chen FF, Zhang HW, Zhang H, Luo S, Huang G, et al. Comparative analysis of the MRI characteristics of meningiomas according to the 2016 WHO pathological classification. Technol Cancer Res Treat. 2020;19:1–9. 10.1177/1533033820983287 PMC7768868 33356976

[8] Upreti T, Dube S, Pareek V, Sinha N, Shankar J. Meningioma grading via diagnostic imaging: a systematic review and meta-analysis. Neuroradiology. 2024;66:1301–10. 38902484 10.1007/s00234-024-03404-0 PMC11246317

[9] Salah F, Tabbarah A, ALArab y N, Asmar K, Tamin H, Makki M, et al. Can CT and MRI features differentiate benign from malignant meningiomas? Clin Radiol. 2019;74:e89815–23. 10.1016/j.crad.2019.07.020 31474303

[10] Bohara M, Nakajo M, Kamimura K, Yoneyama T, Fukukura Y, Kiyao Y, et al. Histological grade of meningioma: prediction by intravoxel incoherent motion histogram parameters. Acad Radiol. 2020;27:342–53. 31151902 10.1016/j.acra.2019.04.012

[11] Hagiwara A, Warntjes M, Hori M, Andica C, Nakazawa M, Kumamaru-Kunishima K, et al. SyMRI of the brain: rapid quantification of relaxation rates and proton density, with synthetic MRI, automatic brain segmentation, and myelin measurement. Invest Radiol. 2017;52:647–57. 28257339 10.1097/RLI.0000000000000365 PMC5596834

[12] Micek M, Aebisher D, Surówka J, Aebisheret-Bartusik D, Madera M. Applications of T1 and T2 relaxation time calculation in tissue differentiation and cancer diagnostics—a systematic literature review. Front Oncol. 2022;12:1010643. 36531030 10.3389/fonc.2022.1010643 PMC9749890

[13] Islim AI, Dona-Kolamunnage R, Mohan M, Moon RDC, Crofton A, Haylock BJ, et al. A prognostic model to personalize monitoring regimes for patients with incidental asymptomatic meningiomas. Neuro Oncol. 2019;142:211–21. 10.1093/neuonc/noz160 PMC7032634 31603516

[14] Elster AD, Challa VR, Gilbert TH, Richardson DN, Contento JC. Meningiomas: MR and histopathologic features. Radiology. 1989;170:857–62. 2916043 10.1148/radiology.170.3.2916043

[15] Kunimatsu A, Kunimatsu N, Kamiya K, Kastura M, Mori H, Ohmoto K. Variants of meningiomas: a review of imaging findings and clinical features. Jpn J Radiol. 2016;34:459–69. 27138052 10.1007/s11604-016-0550-6

[16] Cai Z, Wong YH, So TY. Multisequence magnetic resonance imaging habitat analysis for pre-operative meningioma grade prediction. Quant Imaging Med Surg. 2025;15:7874–84. 40893542 10.21037/qims-2025-1041 PMC12397627

[17] Lee EJ, Kim JH, Park ES, Kim YH, Lee JK, Hong SH, et al. A novel weighted scoring system for estimating the risk of rapid growth in untreated intracranial meningiomas. J Neurosurg. 2017;127:971–80. 28084908 10.3171/2016.9.JNS161669

[18] Häni L, Thomann P, Maragkou T, Vulcu S, Schütz A, Jesse CM, et al. Velocity and pattern of growth of intracranial meningiomas. J Neurosurg. 2025;142:206–13. 39126728 10.3171/2024.4.JNS2446

[19] Wagle PR, Loeschner D, Todorov B, Awuah MY, Lobsien D, Rosahl SK, et al. Proposal of a multiparametric meningioma (MEN-CCVol) score for preoperative discrimination of World Health Organization grade 2/3 from grade 1 intracranial meningiomas based on patient and MRI characteristics. Neurosurgery. 2026;98:221–30. 40459276 10.1227/neu.0000000000003536

[20] Ressel A, Fichte S, Brodhun M, Rosahl S, Gerlach R. WHO grade of intracranial meningiomas differs with respect to patient’s age, location, tumor size and peritumoral edema. J Neurooncol. 2019;145:277–86. 31578671 10.1007/s11060-019-03293-x

[21] Cao T, Jiang R, Zheng L, Zhang R, Chen X, Wang Z, et al. T1 and ADC histogram parameters may be an in vivo biomarker for predicting the grade, subtype, and proliferative activity of meningioma. Eur Radiol. 2023;33:258–69. 35953734 10.1007/s00330-022-09026-5

[22] Li J, Gao X, Nickel MD, Cheng J, Zhu J. Native T1 mapping for differentiating the histopathologic type, grade, and stage of rectal adenocarcinoma: a pilot study. Cancer Imaging. 2022;22:30. 35715848 10.1186/s40644-022-00461-7 PMC9204907

[23] Adams LC, Ralla B, Jurmeister P, Bressem KK, Farlenkamp UL, Hamm B, et al. Native T1 mapping as an in vivo biomarker for the identification of higher-grade renal cell carcinoma: correlation with histopathological findings. Invest Radiol. 2019;54:118–28. 30379727 10.1097/RLI.0000000000000515

[24] Meng T, He N, Liu K, Ke L, Liu H, Zhong L, et al. The diagnostic performance of quantitative mapping in breast cancer patients: a preliminary study using synthetic MRI. Cancer Imaging. 2020;20:88. 33317609 10.1186/s40644-020-00365-4 PMC7737277

[25] Takumi K, Nakanosono R, Nagano H, Hakamada H, Kanzaki F, Kamimura K, et al. Multiparametric approach with synthetic MR imaging for diagnosing salivary gland lesions. Jpn J Radiol. 2024;42:983–92. 38733471 10.1007/s11604-024-01578-4 PMC11364709

[26] Yang F, Li Y, Li X, Yu X, Zhao Y, Li L, et al. The utility of texture analysis based on quantitative synthetic magnetic resonance imaging in nasopharyngeal carcinoma: a preliminary study. BMC Med Imaging. 2023;23:15. 36698156 10.1186/s12880-023-00968-w PMC9875491

[27] Yuan L, Zhao P, Lin X, Yu T, Diao R, Ning G. T1 mapping and reduced field-of‐view DWI at 3.0 T MRI for differentiation of thyroid papillary carcinoma from nodular goiter. Clin Physiol Funct Imaging. 2023;43:137–45. 36440541 10.1111/cpf.12803

[28] Wang F, Yang Q, Zhang Y, Liu J, Liu M, Zhu J. 3D variable flip angle T1 mapping for differentiating benign and malignant liver lesions at 3T: comparison with diffusion weighted imaging. BMC Med Imaging. 2022;22:146. 35982406 10.1186/s12880-022-00873-8 PMC9389795

[29] Cui Y, Han S, Liu M, Wu P, Zhang W, Zhang J, et al. Diagnosis and grading of prostate cancer by relaxation maps from synthetic MRI. J Magn Reson Imaging. 2020;52:552–64. 32027071 10.1002/jmri.27075

[30] Cieszanowski A, Grodzicka-Anysz A, Szeszkowski W, Kaczynski B, Maj E, Gornicka B, et al. Characterization of focal liver lesions using quantitative techniques: comparison of apparent diffusion coefficient values and T2 relaxation times. Eur Radiol. 2012;22:2514–24. 22699872 10.1007/s00330-012-2519-x PMC3472073

[31] Simpkin CJ, Morgan VA, Giles SL, Riches SF, Parker C, deSouza NM. Relationship between T2 relaxation and apparent diffusion coefficient in malignant and non-malignant prostate regions and the effect of peripheral zone fractional volume. Br J Radiol. 2013;86:20120469. 23426849 10.1259/bjr.20120469 PMC3635787

[32] Shao Z, Liu L, Zheng Y, Tu S, Pan Y, Yan S, et al. Molecular mechanism and approach in progression of meningioma. Front Oncol. 2020;10:538845. 33042832 10.3389/fonc.2020.538845 PMC7518150

[33] Cao G, Gao S, Xiong B. Application of quantitative T1, T2 and T2* mapping magnetic resonance imaging in cartilage degeneration of the shoulder joint. Sci Rep. 2023;13:4558. 36941288 10.1038/s41598-023-31644-2 PMC10027866

[34] Mittal S, Pradhan G, Singh S, Batra R. T1 and T2 mapping of articular cartilage and menisci in early osteoarthritis of the knee using 3-Tesla magnetic resonance imaging. Pol J Radiol. 2019;84:e549–64. 32082454 10.5114/pjr.2019.91375 PMC7016502

[35] Fujita S, Yokoyama K, Hagiwara A, Kato S, Andica C, Kamagata K, et al. 3D quantitative synthetic MRI in the evaluation of multiple sclerosis lesions. AJNR Am J Neuroradiol. 2021;42:471–8. 33414234 10.3174/ajnr.A6930 PMC7959431

