# Supplementary material for: Preoperative prediction of meningioma grade based on synthetic MRI quantitative T1 and T2 mapping
Source: Jpn J Radiol. 2026 May 25;44(9):1607–15. doi: 10.1007/s11604-026-02014-5 (PMC13526115; doi:10.1007/s11604-026-02014-5)
Supplement: Supplementary file 1 [file 11604_2026_2014_MOESM1_ESM.docx]

Supplementary Table S1 Comparison of quantitative values between Angiomatous/Microcystic/Metaplastic meningioma and other meningioma

|  | A/M/M | others | *P* value | Cutoff | Sensitivity (%) | Specificity (%) | Accuracy (%) |
| --- | --- | --- | --- | --- | --- | --- | --- |
| Volume (cm^3^) | 11.6 ± 15.8 | 29.2 ± 31.8 | 0.14 | ≤ 0.51 | 80 | 79 | 79 |
| T1 (ms) | 2328.1 ± 375.4 | 1469.8 ± 253 | < 0.001 | > 1862.5 | 100 | 95.8 | 96 |
| T2 (ms) | 175.7 ± 27.9 | 92.8 ± 18.3 | < 0.001 | > 127.1 | 100 | 97.9 | 98 |
| PD (%) | 91.7 ± 1.6 | 89.7 ± 5.2 | 0.19 | > 89.23 | 100 | 47.4 | 50 |
| ADC (10^-3^mm^2^/s) | 1303.8 ± 309.7 | 830.5 ± 103.4 | < 0.001 | > 935.4 | 100 | 89.1 | 89.7 |

Values are expressed as mean ± standard deviation. PD, Proton density; A/M/M, Angiomatous/Microcystic/Metaplastic; ADC, Apparent diffusion coefficient.

Supplementary Figure S1

Scatter plots of T1 and T2 values in Angiomatous/Microcystic/Metaplastic meningioma and other meningioma. A/M/M, Angiomatous/Microcystic/Metaplastic; LGM, Low grade meningioma (WHO grade 1); HGM, High grade meningioma (WHO grade 2 or 3).

Supplementary Figure S2

Receiver operating characteristic curves for discrimination between angiomatous/microcystic/metaplastic meningioma and other meningioma.
